# Neoadjuvant Trastuzumab and Pertuzumab for Early HER2-Positive Breast Cancer: A Real World Experience

**DOI:** 10.1155/2022/7146172

**Published:** 2022-06-30

**Authors:** Benjamin James Hall, Ajay Ashok Bhojwani, Helen Wong, Andrea Law, Helen Flint, Eliyaz Ahmed, Helen Innes, Joanne Cliff, Zaf Malik, Julie Elizabeth O'Hagan, Allison Hall, Rajaram Sripadam, Shaun Tolan, Zulfiqar Ali, Clare Hart, Douglas Errington, Farida Alam, Rosa Giuliani, Shaveta Mehta, Sheena Khanduri, Nicky Thorp, Richard Jackson, Silvia Cicconi, Carlo Palmieri

**Affiliations:** ^1^Department of Molecular and Clinical Cancer Medicine, Institute of Systems, Molecular and Integrative Biology, University of Liverpool, Ashton Street, Liverpool L69 3GE, UK; ^2^The Clatterbridge Cancer Centre NHS Foundation Trust, 65 Pembroke Place, Liverpool L7 8YA, UK; ^3^Liverpool Clinical Trials Centre, University of Liverpool, Liverpool L69 3BX, UK

## Abstract

**Background:**

Randomized studies of neoadjuvant (NA) trastuzumab and pertuzumab combined with chemotherapy for HER2-positive breast cancers (BC) have reported pathological complete response (pCR) rates of 39 to 61%. This study aimed to determine the real-world efficacy and toxicity of NA trastuzumab and pertuzumab combined with chemotherapy in a UK tertiary referral cancer centre.

**Methods:**

HER2-positive early BC patients given neoadjuvant chemotherapy with trastuzumab and pertuzumab between October 2016 and February 2018 at our tertiary referral cancer centre were identified via pharmacy records. Clinico-pathological information, treatment regimens, treatment-emergent toxicities, operative details, and pathological responses and outcomes were recorded.

**Results:**

78 female patients were identified; 2 had bilateral diseases and 48 of 78 (62%) were node positive at presentation. 55 of 80 (71%) tumours were ER-positive. PCR occurred in 37 of 78 (46.3%; 95% CI: 35.3–57.2%) patients. 14 of 23 (60.8%) patients with ER-negative tumours achieved pCR; 23 of 55 (41.8%) were ER-positive and 6 of 19 (31.6%) were ER-positive and PgR-positive. No cardiac toxicity was documented. Diarrhoea occurred in 53 of 72 (74%) patients. Grade 3–4 toxicity occurred in ≥2% patients. These were diarrhoea, fatigue, and infection. The Median follow up period was 45.2 months (95% CI 43.8–46.3) with 71 of 78 (91.0%) remaining disease-free and 72 of 78 (92.3%) alive. Estimated OS at 2 years 86% (95% CI: 75–99%).

**Conclusion:**

This data confirms the efficacy of neoadjuvant chemotherapy combined with dual HER2 directed therapy. While no cardiac toxicity was observed, diarrhoea occurred frequently. The low pCR rate observed in ER and PgR-positive BCs warrants further investigation and consideration of strategies to increase the pCR rate.

## 1. Introduction

In HER2-positive breast cancer neoadjuvant studies have demonstrated that the addition of trastuzumab to chemotherapy improves pathological complete response rates compared to chemotherapy alone [1]. Subsequently, trials employing a number of treatment strategies have investigated the efficacy and tolerability of dual HER2 therapy added to chemotherapy in the neoadjuvant setting, including (1) trastuzumab in combination with chemotherapy versus trastuzumab plus pertuzumab with chemotherapy (NEOSPHERE and PEONY) [2, 3]; (2) different chemotherapy regimens in combination with trastuzumab plus pertuzumab (BERENICE and TRYPHAENA) [4, 5], and (3) trastuzumab plus pertuzumab with chemotherapy versus trastuzumab-emtansine (T-DM1) (KRISTINE) [6].

Primary endpoints varied, with NEOSPHERE, KRISTINE, and PEONY investigating pathological complete response (pCR) rate, while TRYPHAENA and BERENICE assessed the cardiac safety of HER2 blockade (Supplementary Table 1). The definition of pCR as a primary endpoint varied, with NEOSPHERE and PEONY defining it as a complete response in the breast and axilla (ypT0/is/N0), while in NEOSPHERE it was complete response in the breast alone (ypT0/is). The addition of pertuzumab to trastuzumab and chemotherapy in NEOSPHERE and PEONY observed a pCR (ypT0is/N0) rate of 39.3% compared to the control arms of trastuzumab and chemotherapy alone where rates of 21.5% and 21.8% were achieved [2, 3] (Supplementary Table 1). Across all trials, a lower pCR rate was noted for ER-positive tumours (26–57.3%) compared to ER-negative tumours (63.2–79.4%) with the use of the doublet [2–6].

TRYPHAENA and BERENICE found no significant difference in cardiotoxicity between trastuzumab and pertuzumab compared to trastuzumab alone [7, 8]. TRYPHAENA reported symptomatic left ventricular systolic dysfunction (LVSD) in 0.4% of those receiving the doublet and significant left ventricular ejection fraction (LVEF) decline in 3.9–5.6% across all arms of the trial. Similarly, in BERENICE, 1.5% of patients had symptomatic LVSD and 2–6.5% had significant LVEF decline. Diarrhoea is more commonly reported with pertuzumab and trastuzumab, occurring in 28 to 73.5% of patients across trials, with 47–67% requiring pharmacological intervention and 8% resulting in a dose reduction [2–6]. Other toxicities reported with pertuzumab and trastuzumab more prevalent than with trastuzumab alone were neutropenia, alopecia, and nausea [2–6] (Supplementary Table 1).

pCR is associated with improved long-term outcome and the Collaborative Trials in Neoadjuvant Breast Cancer (CTNeoBC) pooled analysis demonstrated that pCR following neoadjuvant chemotherapy combined with trastuzumab was associated with improved long-term outcome in the HER2-positive subgroup irrespective of hormone receptor status (event-free survival (EFS) HR 0.39, (95% CI: 0.31–0.50) and overall survival (HR 0.34, (95% CI: 0.24–0.47) [9]. NEOSPHERE has demonstrated that those achieving pCR had a greater 5-year progression-free survival (PFS) of 85% (95% CI: 76–91) compared to 76% (95% CI: 71–81) in those that did not (HR: 0.54, 95% CI: 0.29–1.00) [10]. A pooled analysis of EFS across several the randomized HER2 doublet trials also demonstrated reduction in the risk of an event in those achieving a pCR (HR: 0.33; 95% CI: 0.25–0.43) [11].

Given the known differences between the clinical trial environment and routine clinic setting, we sought to explore the efficacy and toxicity of neoadjuvant HER2 doublet in combination with chemotherapy within the practice of a tertiary referral NHS cancer centre in the UK.

## 2. Patients and Methods

### 2.1. Patient Population

Patients with HER2 positive early breast cancer, treated with neoadjuvant chemotherapy in combination with trastuzumab and pertuzumab, between October 2016 and January 2018 via the Cancer Drugs Fund (CDF) at our tertiary cancer referral centre were identified via a search of pharmacy records. Patients receiving at least one cycle of neoadjuvant trastuzumab and pertuzumab were eligible. Patients presenting with de novo metastatic disease were excluded. This project was approved as an audit by the local clinical audit subcommittee (Ref Number 1718–45).

### 2.2. Data Collection and Analysis

The clinicopathological data collected included patient age, tumour information at time of diagnosis including radiological size, histological grade on core biopsy, histological subtype, axillary nodal status on biopsy, and estrogen receptor status. Assessment of progesterone receptor status was not performed in all cases as this is not considered mandatory, particularly in those of ER-status [12]. In the presence of multifocal disease within the same breast, total tumour size (T stage) was determined by the diameter of the largest lesion according to radiographic findings. In patients found to have bilateral disease each tumour was treated separately. Posttreatment information collected included date and type of surgery, histopathological information including size, grade, and axillary lymph node status. Echocardiogram reports were reviewed to assess LVEF. Details of the neoadjuvant chemotherapy regimen and temporal relationship to the initiation of trastuzumab-pertuzumab were collected.

### 2.3. HER2 Positivity

All core biopsy reports from referring units were reviewed to confirm HER2 overexpression, as confirmed in a histopathological report by immunohistochemistry (IHC) and/or fluorescence in situ hybridization (FISH).

### 2.4. Treatment Regimens

Treatment regimens consisted of (1) FEC-THP: three cycles of FEC100 (fluorouracil, epirubicin, cyclophosphamide) every 21 days followed by four cycles of docetaxel, trastuzumab, and pertuzumab. Where there were allergic reactions to docetaxel or there were tolerability issues, patients were switched to paclitaxel weekly. (2) AC/EC-THP: three cycles of either EC (epirubicin, cyclophosphamide) every 21 days or AC (adriamycin, cyclophosphamide) followed by four cycles of docetaxel, trastuzumab, and pertuzumab (3) TCHP: docetaxel, carboplatin, trastuzumab, and pertuzumab. Neoadjuvant THP, followed by FEC postoperatively, was also delivered. Regimen dosing is described in Supplementary Figure 1.

Granulocyte colony stimulating factor (Filgrastim), postoperative radiotherapy and/or endocrine therapy as well as adjuvant zoledronic acid were prescribed as per local guidelines.

### 2.5. Study End-Point Definitions

Pathological complete response was defined as the complete absence of invasive and in situ disease in the breast and the axillary node (ypT0/is N0). For non-pCRs, residual tumour size was based on maximal diameter as described in the pathological report.

An event was defined as: ipsilateral invasive breast tumour recurrence, local/regional invasive breast cancer recurrence, distant recurrence, or death from any cause, whichever occurs first. Progression-free survival (PFS) was measured from date of the diagnostic core biopsy to first event, while disease-free survival (DFS) was measured from the date of definitive surgery to the first event. Patients who had not experienced an event were censored at the date of last follow-up when they were known to be event-free. Overall survival (OS), measured from the date of the diagnostic core biopsy to death due to any cause. Survivors were censored at the date they were last known to be alive. The data cut-off was 6^th^ March 2021.

### 2.6. Adverse Events

Safety outcome measures included type, incidence, and severity of adverse events according to the Common Terminology Criteria for Adverse Events (CTCAE) criteria v1.1, documented by nursing staff at each treatment delivery visit.

Cardiotoxicity was defined as a decrease in LVEF of 10% from baseline or a decrease to an LVEF of less than 55% at any time, as well as any Class 3 or 4 congestive heart failure according to the New York Heart Association [13].

### 2.7. Statistical Analysis

Continuous variables are presented with their median and interquartile range (IQR), while categorical variables are described as frequency counts and proportion percentages. Fisher exact or Wilcoxon–Mann–Whitney tests were performed as appropriate to compare proportions and distributions. PCR was also shown graphically with bar plots and related 95% confidence intervals.

## 3. Results

Between October 2016 and February 2018, 79 patients received at least one dose of neoadjuvant trastuzumab and pertuzumab; clinico-pathological information is summarized in Table 1. One patient was found to have de novo metastatic disease and was therefore excluded, leaving 78 patients. All patients were female with a median age of 50 years (IQR: 44.4–60.2) and a median BMI of 27 kg/m^2^, (IQR: 23.2–29.4). 13 (17%) of 78 patients were less than 40 years of age. Most cases, 67 of 78 (86%), presented symptomatically. The median tumour size at presentation was 30 mm (IQR: 23.0 to 47.5) with 19 of 78 (24.4%) having multifocal disease. 4 of 78 (4.9%) were inflammatory BC. Two patients had bilateral disease, thus a total of 80 tumours were assessable for pathological assessment. 55 of 80 (68.8%) tumours were ER positive; PgR staining was performed in 33 of 55 (60%). 19 of 33 (58%) were PgR positive and 14 of 33 (42%) were PgR negative. 23 of 80 (28.8%) were confirmed ER-negative and PgR-negative. In patients with bilateral tumours, 1 had bilateral HER2 positive, ER positive and PgR-positive tumours while the other had 1 HER2 positive, ER positive and PgR positive and the other triple negative.

61 of 80 (76.3%) assessable axillae were biopsied at diagnosis. Of these, 5 of 61 (8%) had sentinel lymph node biopsy prior to commencing systemic therapy. 48 of 61 (86%) revealed axillary node involvement, with 1 patient having bilateral axillary node involvement, giving a total of 49 of 80 (61%) axillae involved. Therefore, 49 of 78 (65.4%) patients had pathologically confirmed lymph node involvement at presentation.

### 3.1. Treatment Regimens

63 of 78 (81%) patients were commenced on FEC-THP, 7 of 78 (9.0%) received EC/AC-HP and 8 of 78 (10.3%) TCHP. 3 of 78 (3.8%) received neoadjuvant THP with postoperative FEC (Supplementary Figure 1). Both patients with bilateral disease received FEC-THP.

21 of 78 (26.9%) patients switched from docetaxel to paclitaxel over the course of treatment due to toxicity. Of the patients switched to paclitaxel, 1 received AC/EC-THP, 1 TCHP, and 19 FEC-THP (30.2%); 1 (5.3%) of these 19 patients subsequently switched to nab-paclitaxel.

The median time from diagnosis to treatment initiation was 4.9 weeks (IQR: 3.9–6.1). 11 of 78 (14%) patients did not receive all doses of HER2 therapy due to toxicity, proceeding to surgery. No patients discontinued treatment due to disease progression. Supplementary Figure 2 summarizes regimen alterations, dose reductions, and deferrals.

### 3.2. Surgical Details

All 78 patients underwent surgery. The median time from diagnosis to surgery was 27.5 weeks (IQR: 24.8–29.3). 39 of 78 (50%) patients underwent mastectomy; both patients with bilateral disease underwent bilateral mastectomies giving a total of 41 mastectomies. The remaining 39 (50%) patients had breast conserving surgery. Regarding the axilla, 36 of 78 (45%) underwent axillary node clearance (ANC), while sentinel lymph node biopsy (SLNB) was performed in 46 of 78 (57.5%), including 4 cases performed up-front. Two patients had both SLNB and ANC; hence the total number of axillary procedures was 82. Surgical details are summarized in Table 1.

### 3.3. Pathological Response Data

Pathological complete response (ypT0is/N0) was observed in 37 of 80 (46%; 95% CI: 35–57%) assessable breast cancers, representing 37 of 78 (47%: 95% CI: 36–58.5%) patients. In 5 of 80 tumours (6%; 95%CI: 1–12%), pCR was achieved in the breast alone. Of the 2 patients with bilateral disease, 1 achieved unilateral pCR in the breast while the other, with a triple negative tumour, did not achieve pCR in either breast or axilla. 21 of 78 patients (27%; 95% CI: 17–36%) had residual nodal disease at surgery.

**Figure 1:**
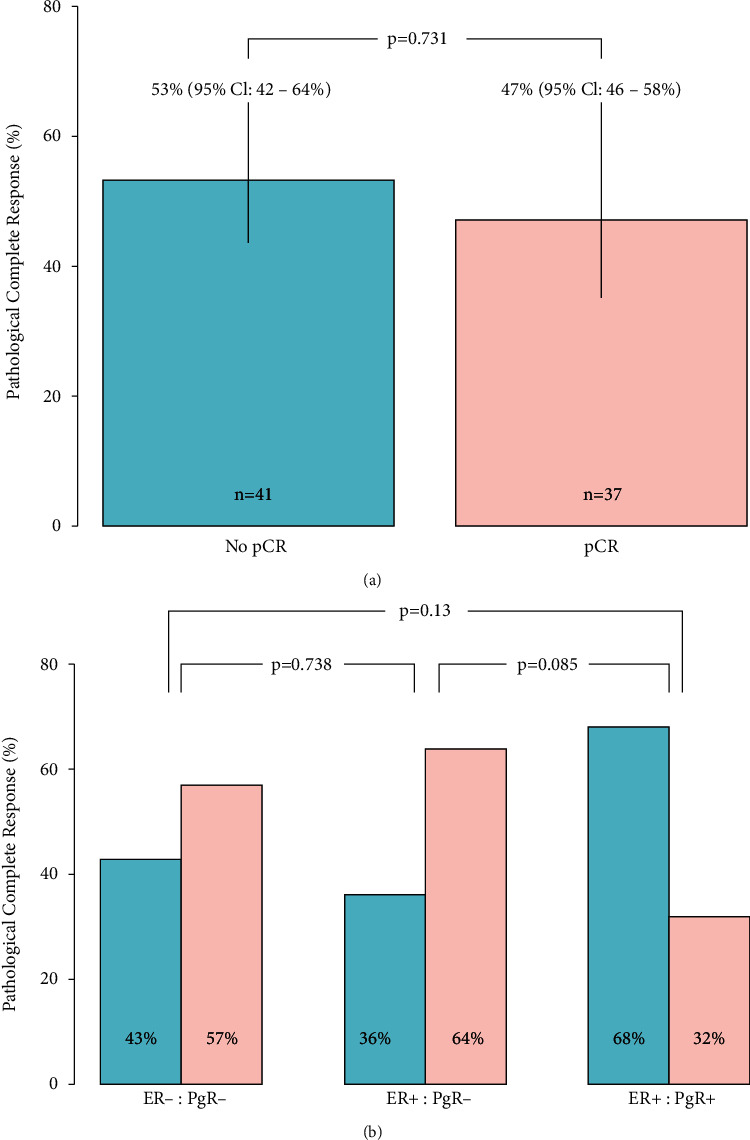
Pathological complete response rate by hormone receptor status. (a) Overall pCR rate (ypT0/is, N0) rate (b) pCR rate based on ER and PgR status of tumours where available. ER: estrogen receptor; PgR: progesterone receptor.

23 of 55 (42%; 95% CI: 29–55%) of the ER-positive tumours achieved pCR as compared to 14 of 23 (61%; 95% CI: 41–81%) that were ER-negative (*p*=0.198) (Figure 1). PCR was achieved in 9 of 14 (64%; 95% CI: 39–89%) tumours which were ER-positive, and PgR-negative compared to 6 of 19 (32%; 95% CI: 11–52.5%) which were ER-positive, PgR-positive (*p*=0.11) (Figure 1). Clinicopathological details of those where pCR was not achieved are summarized in Supplementary Table 2. Information on pCR by treatment regimen and age is presented in Supplementary data.

### 3.4. Outcome Data

At a median follow-up of 45.2 months (95% CI: 44–46%) 72 of 78 (92%) patients remained alive and disease free. 6 of 78 (6%) patients suffered disease recurrence. Of these, 4 of 6 (66%) had not achieved pCR (Supplementary Table 3). 1 patient developed a rectal cancer during follow up.

**Figure 2:**
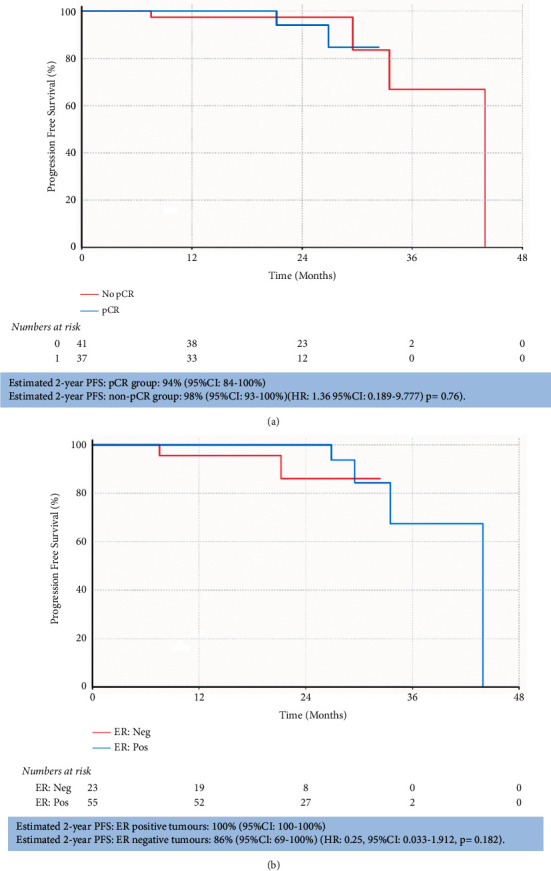
Progression-free survival Kaplan–Meier curves. Progression-free survival is stratified by pCR status (a) and ER status (b). Estimated 2-year PFS in each subgroup is provided with respective hazard ratios and *p* values.

Of those patients who achieved pCR, 2 of 37 (5%) had a progression-free event compared to 4 of 41 (10%) patients who did not. 2-year PFS rates were 94% (95% CI: 84–100%) for patients who achieved pCR, compared to 98% (95% CI: 93–100%) where pCR was not achieved (HR: 1.36; 95% CI: 0.189–9.777); (*p*=0.760) (Figure 2(a)). Overall 2-year PFS rate was 95% (95% CI: 89–100%) (Figure 2(b)). PFS data by ER and PgR status are summarized in Table 2. Disease-free survival results were consistent with PFS (Table 2 and Supplementary Figure 4).

**Figure 3:**
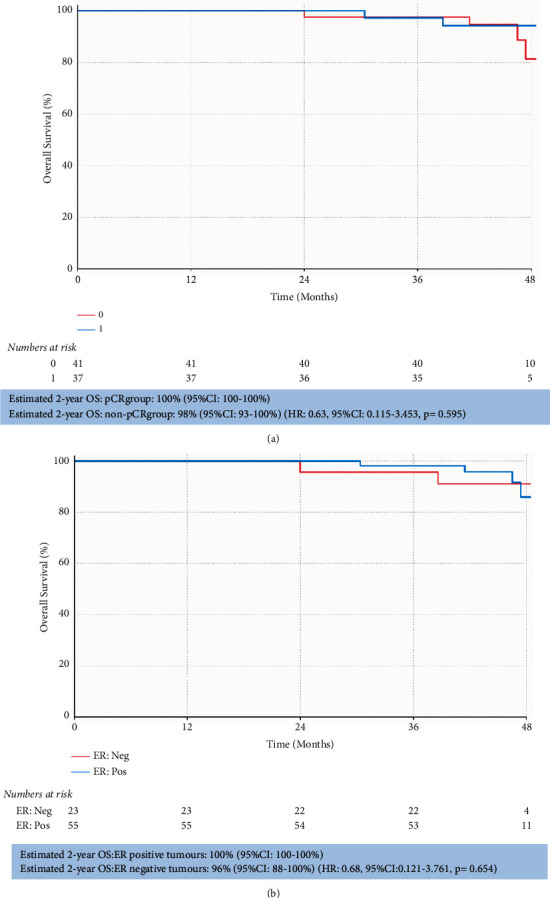
Overall survival Kaplan–Meier curves. Overall survival is stratified by pCR (a) and ER status (b). Estimated 2-year OS in each subgroup is provided with respective hazard ratios and *p* values.

The median OS was not reached. Estimated OS at 2 years was 86% (95%CI: 75–99). At 2 years in those patients who achieved pCR OS, the rate was 100% (95% CI: 100–100%) and 98% (95% CI: 93–100) in the non-pCR group (HR: 0.63 (95% CI: 0.115–3.453); *p*=0.595) (Figure 3). OS data by ER and PgR status are summarized in Table 2.

### 3.5. Toxicity

All patients experienced toxicity, with 11 of 78 (14%) experiencing grade 3 or higher. The most frequently occurring toxicity was fatigue, occurring in 71 of 72 (99%) patients. The most frequently occurring grade 3 or higher toxicity was infection, occurring in 4 of 72 (5.5%) patients, leading to 3 (75%) of these patients ceasing treatment early. Diarrhoea was documented in 53 of 72 (73.5%) patients, occurring at grade 3 or higher in 2 of 72 (3%) patients. The most common treatment-related toxicities are summarized in Table 3.

### 3.6. Cardiac Safety

71 of 78 (91%) patients underwent baseline echocardiograms. 15 of 71 (21%) had baseline scans only, 28 of 71 (39%) patients had one scan after treatment initiation and the remaining 28 of 71 (39%) patients had two or more during neoadjuvant and adjuvant treatment. 35 of 78 (45%) patients received a scan every 4 months as recommended. No patients developed a significant decline in LVEF or symptomatic LVSD from the initiation of neoadjuvant treatment until last follow-up, with a mean change of <1% in LVEF in those measured.

## 4. Discussion

In the neoadjuvant setting, chemotherapy in combination with trastuzumab and pertuzumab in HER2 positive early BC patients is associated with an increase in the pCR rate compared to trastuzumab alone, with the doublet resulting in rates of 39.3 to 60.7% [2–6]. It is recognized that the real-world setting differs from that of randomized trials and that there is a need to develop real-world data regarding novel agents [14].

The cohort identified was prospectively treated between October 2016 and June 2018, reflecting the initial period when neoadjuvant trastuzumab and pertuzumab were made available to patients in England via the CDF. Our population of early HER2-positive BC patients was broadly consistent with those in pivotal trials. We report a median age of 50 years; 68.8% of tumours being hormone receptor-positive and 86% LN positive, as compared to a median age of 49–50 years, hormone receptor-positive 48–62% and LN positive 70–76% in pivotal studies [2–6].

pCR (ypT0/is/N0) occurred in 46% (37 of 80) of tumours in our cohort; within the range of 39.3 to 60.7% reported by previous trials [2–6]. Several other real-world studies (RWS) have investigated the impact of neoadjuvant trastuzumab and pertuzumab. These have varied in size, 57–254 patients, as well as in the chemotherapy backbone utilized pCR rates ranged from 58.4% to 76.1% (Supplementary Table 5) [15–21].

We report a higher pCR rate for ER-negative as compared to ER-positive BCs, in keeping with the published randomized studies which ranged from 63.2–79.4% in ER-negative compared to 26–57.3% in ER-positive. [2–6]. As well as RWS, where pCR rates in HR negative tumours ranged from 70.8% to 80.9% compared to 52.3% to 55.4% in those that were HR positive [15–21]. ER status was reported as a significant predictor of pCR on multivariate analysis in some studies [17, 19].

We investigated the efficacy of trastuzumab and pertuzumab in relation to the ER and PgR status of tumours and found a pCR rate of 64.3% (95%CI: 39–89%) in ER-positive and PgR-negative tumours compared to 31.6% (95%CI: 11–52.5%) for ER-positive and PgR-positive tumours. Across all five pivotal trials, “positive HR status” is defined as either ER or PgR positivity, with no data reported for ER-positive/PgR-positive and ER-positive/PgR-negative tumours. Across RWS, two reported PgR status of tumours [18, 19]; one of these found PgR status was a significant predictor of pCR on univariate analysis (*p* < 0.01) but not on multivariate analysis [19]. The absolute pCR rate according to PgR status was not reported.

The lower pCR rate reported with chemotherapy and HER2 doublet in ER-positive/PgR-positive BCs raises questions regarding the optimal treatment backbone for such BCs. In the NA-PHER2 study, 30 ER-positive and HER2-positive breast cancers were treated with 18 weeks of trastuzumab and pertuzumab, combined with fulvestrant and palbociclib and were assessed for pCR (ypT0/is/N0) [22]. NA-PHER2 reported a pCR rate of 27% (95% CI: 12–46%) [22]. In the PerELISA study, a pCR rate of 20.5% (95%CI: 11.1–34.5%) following 15 weeks of trastuzumab, pertuzumab, and letrozole was reported [23]. None of these studies reported any data based on the PgR status. These data demonstrate that a noncytotoxic approach combining HER2-directed and endocrine therapy with or without a CDK4/6 inhibitor might be an effective and less toxic treatment option for ER-positive and PgR-positive BCs in the neoadjuvant setting.

We reported excellent outcomes in our patient group with 12 and 24-month PFS estimates of 99% (95% CI 96–100%) and 95% (95% CI 89–100%). This is consistent with the data reported from arms of pivotal studies for patients who received HER2 doublet in combination with chemotherapy with NEOSPHERE reporting a 5-year PFS of 86% (95% CI: 77–91%) [10]; TRYPHAENA a 3-year PFS of 87–89% across the three arms [24], and KRISTINE having a 3-year event-free survival rate of 94.2% [25].

Since March 2019, within England, adjuvant trastuzumab and pertuzumab have been for patients with node-positive disease or axillary node scarring consistent with a response to treatment. While we did not examine for axillary node scarring, 21 of 41 (51.2%) patients that did not achieve pCR had residual nodal disease and would therefore have been eligible to have continued treatment. Similarly, adjuvant TDM-1 which has demonstrated a 50% lower risk of recurrence in those with residual disease at surgery [26] has also subsequently become available for use within England on the NHS, and 41 of 78 (63%) of patients in the current study where pCR was not achieved would have been eligible for T-DM1.

No cardiotoxicity was identified in our population as defined by either LVEF decline of ≥10% or symptom onset. These data are reassuring as to the safety of HER2 therapy in the context of a real population. The lack of cardiotoxicity may reflect the fact that clinicians have been using HER2 directed therapy for some time and so are better equipped at selecting patients as well as optimizing cardiac function in those with pre-existing conditions in conjunction with cardio-oncology colleagues [27].

Diarrhoea of any grade was very common in our cohort, occurring in 73.6% of patients (Table 3), with only 2.6% experiencing grade 3 or higher, as compared to rates of 10.1%–73.5% and 0–15% for all grades and grade 3 or higher, respectively, from previous studies (Supplementary Table 4) [2–6]. While 14.1% patients did not receive all doses of HER2 directed therapy, in none of these instances was diarrhoea the cause of early treatment cessation. These data also highlight the challenges of using docetaxel in the real world as demonstrated by the 27% (21 of 78) of patients who were switched to weekly paclitaxel or nab-paclitaxel.

### 4.1. Limitations

This study has several limitations. Firstly, this data is retrospective. Although patients were identified from a prospective database, the population size is relatively small and is therefore inadequately powered for robust analysis between subgroups such as ER and PgR status. Furthermore, since this cohort began treatment, several alternative therapeutic options have become available for use in early HER2 BC including postoperative pertuzumab and adjuvant TDM-1 [26, 28]. Therefore, the outcome data reported here may differ from those of patients diagnosed more recently. Finally, the follow-up time for this cohort remains relatively short, and to perform true survival outcome analysis a longer follow-up interval is required.

## 5. Conclusion

In conclusion, in the context of a tertiary referral NHS Cancer Centre neoadjuvant chemotherapy in combination with trastuzumab and pertuzumab is a safe and efficacious treatment for early HER2 breast cancer. These data are in keeping with data from randomized controlled studies and similar RWS, however the lower pCR rate in ER-positive, PgR-positive tumours identified here warrants further investigation.

## Figures and Tables

**Table 1:** Clinicopathological characteristics at diagnosis and details of final breast and axillary surgery. Gender is not displayed as all patients identified were female. The number of patients having axillary intervention amounts to greater than 100%, as 2 patients had sentinel lymph node biopsy as well as ANC. Axillary lymph node dissection.

Characteristic	Frequency (*n*, (%))
Median age (yrs) (IQR)	50 (44.4–60.2)
Grouped ages *n* (%)	
<35 yrs	5 (6.4)
35–50 yrs	33 (42.3)
51–65 yrs	29 (37.2)
>65 yrs	11 (14.1)
Median BMI (kg/m^2^) (IQR)	27 (23.2–29.4)
Postmenopausal *n* (%)	38 (49)
ECOG Performance score *n* (%)	
0	66 (85)
1	12 (15)
Tumour laterality	
Left	41 (51)
Right	39 (49)
Median tumour size (mm) (IQR)	30 (23.0–47.5)
T stage (mm)	
T1; 0–20 mm	11 (13.8)
T2; 20–50 mm	54 (67.5)
T3; >50 mm	15 (18.8)
Nodal status *n* (%):	
Positive	52 (65.0)
Negative	28 (35.0)
Histological subtype *n* (%):	
Ductal	74 (92.5)
Lobular	2 (2.5)
Other	4 (5.0)
Inflammatory breast cancer	4 (5.0)
Histological grade (core biopsy) *n* (%)	
1	2 (2.5)
2	31 (38.8)
3	47 (58.8)
Receptor status	
ER+, PgR+	19 (23.8)
ER+, PgR−	14 (17.5)
ER+, PgR unknown	23 (28.8)
ER−, PgR−	23 (28.8)
ER−, PgR unknown	1 (1.3)
Breast surgery	
Mastectomy	41 (51.3)
Breast conservation	39 (48.7)
Axillary surgery	
Sentinel lymph node biopsy	46 (57.5)
Axillary lymph node dissection	36 (45.0)

**Table 2:** Estimated survival outcomes according to hormone receptor and pCR status (^*∗*^unobtainable).

Outcome status	Progression-free survival			Disease-free survival			Overall survival		
	24-month estimate (95% CI)	Hazard ratio (95% CI)	*p* value	24-month estimate (95% CI)	Hazard ratio (95% CI)	*p* value	24-month estimate (95% CI)	Hazard ratio (95% CI)	*p* value
ER-positive ER-negative	100 (100–100) 86 (69–100)	0.25 (0.033, 1.912)	0.182	94 (84–100) 85 (66–100)	0.41 (0.067, 2.509)	0.336	100 (100–100) 96 (88–100)	0.68 (0.121, 3.761)	0.654
PgR-positive	100 (100–100)	Unob.^*∗*^	Unob.^*∗*^	100 (100–100)			100 (100–100)		
PgR-negative	92 (81–100)			81 (61–100)			97 (92–100)		
pCR No pCR	94 (84–100) 98 (93–100)	1.36 (0.189, 9.777)	0.76	83 (63–100) 98 (93–100)	1.5 (0.231, 9.713)	0.672	100 (100–100) 98 (93–100)	0.63 (0.115, 3.453)	0.595

**Table 3:** Summary of the ten most frequently reported treatment related toxicities.

Adverse effect	Total (all grades) *n* (%)	Grade 3 or 4 *n* (%)
Alopecia	69 (95.8)	0
Fatigue	71 (98.6)	2 (2.6)
Nausea	49 (68.1)	0
Vomiting	15 (20.8)	1 (1.3)
Diarrhoea	53 (73.6)	2 (2.6)
Constipation	41 (56.9)	1 (1.3)
Mucositis	50 (69.4)	1 (1.3)
Palmar-planter syndrome	23 (31.9)	0
Peripheral neuropathy	40 (55.6)	0
Infection	40 (55.6)	4 (5.2)

## Data Availability

Data in this article is not stored in a publicly archived dataset due to the confidential nature.

## Supplementary Materials

Supplementary Materials Supplementary Table 1: current randomized trial data examining dual HER2 blockade and chemotherapy in the neoadjuvant setting for early HER2 positive BC. T: docetaxel, H: trastuzumab, P: pertuzumab, FEC: 5-FU/epirubicin/cyclophosphamide, TCHP: docetaxel/carboplatin/trastuzumab/pertuzumab, p: paclitaxel, T-DM1: trastuzumab-emtansine, plac: placebo. DFS: disease-free survival, OS: overall survival. Approximate values are given in circumstances where exact values are not attainable and were therefore determined from a graphical representation of the data. Supplementary Figure 1: neoadjuvant dosage regimen for locally invasive or inflammatory HER2 positive breast cancer patients at The Clatterbridge Cancer Centre NHS Foundation Trust, Liverpool. Supplementary Figure 2: neoadjuvant chemotherapy regimens, dose reductions, and deferrals. AC: doxorubicin and cyclophosphamide, FEC: 5-fluorouracil, epirubicin, and cyclophosphamide, TCHP: docetaxel, carboplatin, trastuzumab + pertuzumab, THP = docetaxel, trastuzumab, pertuzumab. NB: 2 patients received THP prior to neoadjuvant FEC, while 3 patients received THP alone prior to surgery, 1 of whom went on to receive FEC adjuvantly. Supplementary Table 2: characteristics of residual tumour in those patients who did not achieve pathological complete response. Samples insufficient for assessment were either too small (*n* = 3), of inadequate quality (*n* = 2) or were found to only have intravascular tumour emboli remaining in the breast (*n* = 1). Supplementary Figure 3: progesterone receptor status outcome data. Kaplan–Meier curves demonstrate (A) progression-free survival and (B) overall survival in PgR-positive and PgR-negative tumours. Estimated 2-year PFS and OS according to PgR status are provided below (hazard ratios and *p* values were unobtainable for PgR data). Supplementary Figure 4: disease-free survival Kaplan–Meier curves. Disease-free survival is stratified by (A) pCR status and (B) hormone receptor status. Estimated 2-year OS in each subgroup is provided with respective hazard ratios and *p* values. Supplementary Table 3: summary of patients who developed recurrent disease and the treatments they received; ^*∗*^All the patients have died; ^*∗∗*^found to be triple negative on biopsy of recurrence; AC: adriamycin, cyclophosphamide; RT; radiotherapy. Supplementary Table 4: adverse events reported by published neoadjuvant and dual HER2 blockade trials. Rates reported include adverse events of all severity. Rates reported in this paper are documented in the far-right column. ^*∗*^5.2% of patients in this cohort presented with either a Grade 3 or 4 infection during their course of treatment. Supplementary Table 5: summary of real-world studies examining the efficacy of neoadjuvant chemotherapy and pertuzumab/trastuzumab doublet. ^*∗*^Only overall pCR rates were reported in some papers but were not stratified according to both hormone receptor status and treatment regimen. NAC: neoadjuvant chemotherapy (taxanes with or without anthracyclines; regimen not specified). A: doxorubicin C: carboplatin; H: trastuzumab; P: pertuzumab; T: docetaxel Cy: cyclophosphamide Pa: paclitaxel NR: results were not specifically reported.
