# Supplementary material for: Neoadjuvant Trastuzumab and Pertuzumab for Early HER2-Positive Breast Cancer: A Real World Experience
Source: Breast J. 2022 Jun 30;2022:7146172. doi: 10.1155/2022/7146172 (PMC9262537; doi:10.1155/2022/7146172)
Supplement: Supplementary Materials — Supplementary Table 1: current randomized trial data examining dual HER2 blockade and chemotherapy in the neoadjuvant setting for early HER2 positive BC. T: docetaxel, H: trastuzumab, P: pertuzumab, FEC: 5-FU/epirubicin/cyclophosphamide, TCHP: docetaxel/carboplatin/trastuzumab/pertuzumab, p: paclitaxel, T-DM1: trastuzumab-emtansine, plac: placebo. DFS: disease-free survival, OS: overall survival. Approximate values are given in circumstances where exact values are not attainable and were therefore determined from a graphical representation of the data. Supplementary Figure 1: neoadjuvant dosage regimen for locally invasive or inflammatory HER2 positive breast cancer patients at The Clatterbridge Cancer Centre NHS Foundation Trust, Liverpool. Supplementary Figure 2: neoadjuvant chemotherapy regimens, dose reductions, and deferrals. AC: doxorubicin and cyclophosphamide, FEC: 5-fluorouracil, epirubicin, and cyclophosphamide, TCHP: docetaxel, carboplatin, trastuzumab + pertuzumab, THP = docetaxel, trastuzumab, pertuzumab. NB: 2 patients received THP prior to neoadjuvant FEC, while 3 patients received THP alone prior to surgery, 1 of whom went on to receive FEC adjuvantly. Supplementary Table 2: characteristics of residual tumour in those patients who did not achieve pathological complete response. Samples insufficient for assessment were either too small (n = 3), of inadequate quality (n = 2) or were found to only have intravascular tumour emboli remaining in the breast (n = 1). Supplementary Figure 3: progesterone receptor status outcome data. Kaplan–Meier curves demonstrate (A) progression-free survival and (B) overall survival in PgR-positive and PgR-negative tumours. Estimated 2-year PFS and OS according to PgR status are provided below (hazard ratios and p values were unobtainable for PgR data). Supplementary Figure 4: disease-free survival Kaplan–Meier curves. Disease-free survival is stratified by (A) pCR status and (B) hormone receptor status. Estimated 2-year O [file 7146172.f1.docx]

| **Variable** | **Study** | | | | | **Current study** |
| --- | --- | --- | --- | --- | --- | --- |
| **Trial name** | NEOSPHERE | TRYPHAENA | BERENICE | KRISTINE | PEONY |  |
| **Authors** | Gianni et al., 2012 | Schneeweiss et al.,2013 | Swain et al., 2017 | Hurvitz et al, 2018 | Zhimin et al, 2019 | Hall et al 2019 |
| **No of women** | 107 | 225 | 397 | 221 | 329 | 78 |
| **Design** | Randomised, open-label, multicenter, Phase 2 | Randomised, open-label, multicenter, multinational, Phase 2 | Non-randomised, open-label, multicenter, Phase 2 | Randomised, open-label, multicenter, Phase 3 | Randomised, multicenter, double-blind, placebo-controlled phase 3 trial | Single centre, retrospective analysis |
| **Primary endpoint** | pCR | LVEF decline >10% from baseline, LVSD | LVEF decline >10% from baseline, LVSD | pCR | pCR | pCR |
| **pCR definition** | Breast alone (ypT0/is) | Breast alone (ypT0/is) | Breast and nodes (ypT0/is, ypN0) | Breast and nodes (ypT0/is, ypN0) | Breast and nodes (ypT0/is, ypN0) | Breast and nodes (ypT0/is, ypN0) |
| **Treatment given** | 1) THP  2) TH  3) TP  4) HP | 1) FEC+THP  2) FEC -> THP  3) TCHP | 1) FEC -> THP  2) ddAC -> pHP | 1) TCHP  2) T-DM1 + P | 1) THP  2) Plac. + HP | 1) FEC-THP  2) FEC-PHP  3) TCHP |
| **pCR rate**  **(ypT0/is)** | 1) 45.8%  2) 29.0%  3) 24.0%  4) 16.8% | 1) 61.6%  2) 57.3%  3) 66.2% | 1) not reported  2) not reported | 1) not reported  2) not reported | 1) not reported  2) not reported | 1) 61.4%  2) 65.0%  3) 22.2% |
| **pCR rate**  **(ypT0/is, ypN0)** | 1) 39.3%  2) 21.5%  3) 17.7%  4) 11.2% | 1) 50.7%  2) 45.3%  3) 51.9% | 1) 60.7%  2) 61.8% | 1) 56%  2) 44% | 1) 39.3%  2) 21.8% | 1) 52.2%  2) 65.0%  3) 22.2% |
| **Hormone receptor positive pCR** | 1) 26.0%  2) 20.0%  3) 17.4%  4) 5.9% | 1) 46.2%  2) 48.6%  3) 50.0% | 1) 57.3%  2) 51.6% | 1) approx. 43%  2) approx. 35% | 1) approx. 45%  2) approx..19% | Overall: 58.3% |
| **Hormone receptor negative pCR** | 1) 63.2%  2) 36.8%  3) 30.0%  4) 27.3% | 1) 79.4%  2) 65.0%  3) 83.8% | 1) 68.0%  2) 81.5% | 1) approx. 71%  2) approx. 52% | 1) approx. 35%  2) approx. 20% | Overall: 41.1% |
| **Outcome data** | 5-year DFS: 81% | 3-year PFS: 87-89% | Not reported | 3-year EFS: 94.2% | Not reported | Estimated 2-year OS: 86% |
| **Median followup** (months) | 36 (not in original report) | 21 | 14.5 | 36 (not in original report) | Not reported | 45.2 |
| **Table 1.** Current randomized trial data examining dual HER2 blockade and chemotherapy in the neoadjuvant setting for early HER2 positive BC. T: docetaxel, H: trastuzumab, P: pertuzumab, FEC: 5-FU/epirubicin/cyclophosphamide, TCHP: docetaxel/carboplatin/trastuzumab/pertuzumab, p: paclitaxel, T-DM1: trastuzumab-emtansine, plac: placebo. DFS: disease free survival, OS: overall survival. Approximate values are given in circumstances where exact values were not attainable and were therefore determined from graphical representation of data. | | | | | | |

**Supplementary**

3 x FEC/EC

HER2 positive locally advanced, inflammatory or early breast cancer patients

6 X TCHP

4 x D-HP

Surgery

14 Cycles Trastuzumab +/- endocrine therapy +/- RT

**Fluorouracil** 500mg/m^2^, **Epirubicin** 90mg/m^2^, **Cyclophosphamide** 500mg/m^2^

**Docetaxel** 75mg/m^2^ loading, 100mg/m^2^ maintenance. **Trastuzumab** 8mg/kg loading, 6mg/kg maintenance. **Pertuzumab** 840mg loading, 420mg maintenance.

**Docetaxel** 75mg/m^2^, **Carboplatin** AUC 6, **Trastuzumab** 8mg/kg loading, 6mg/kg maintenance. **Pertuzumab** 840mg loading, 420mg maintenance.

12 x P-HP

**Paclitaxel** 80mg/m^2^.

**Trastuzumab** 8mg/kg loading, 6mg/kg maintenance. **Pertuzumab** 840mg loading, 420mg maintenance.

**Supplementary Figure 1**. Neoadjuvant dosage regimen for locally invasive or inflammatory HER2 positive breast cancer patients at The Clatterbridge Cancer Centre NHS Foundation Trust, Liverpool.


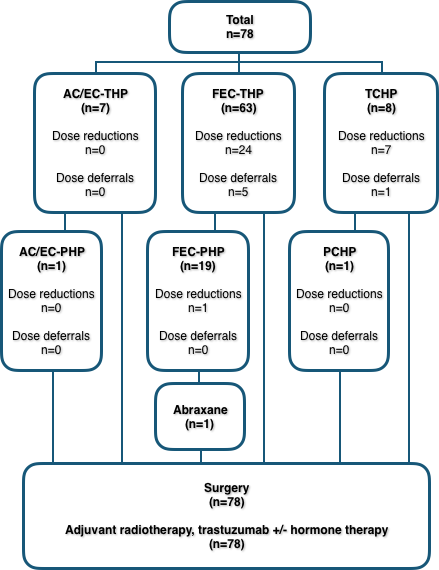


**Supplementary Figure 2.** **Neoadjvuant chemotherapy regimens, dose reductions and deferrals**

AC: doxorubicin and cyclophsophomide, FEC: 5-fluorouracil, epirubicin and cyclophosphamide, TCHP: docetaxel, carboplatin, trastuzumab + pertuzumab, THP =docetaxel, trastuzumab, pertuzumab. NB: 2 patients received THP prior to neoadjuvant FEC, while 3 patients received THP alone prior to surgery; 1 of whom went on receive FEC adjuvantly.

| **Variable** | **Residual breast disease characteristics**  **(n = 37, (%))** |
| --- | --- |
| **Median tumour size (mm)** (IQR) | **13** (6 – 22) |
| **Grouped tumour size (mm):** |  |
| 0-20 mm | **25** (71.0) |
| 20-50 mm | **7** (22.6) |
| >50 mm | **5** (6.5) |
| **Histological grade:** |  |
| 1 | **4** (5.9) |
| 2 | **23** (50.0) |
| 3 | **5** (17.6) |
| *Samples insufficient for assessment* | **5** (14.7) |
| *Variable* | **Residual nodal disease characteristics**  **(n=41, (%))** |
| **Lymph node status:** |  |
| Positive | **21** (51.2) |
| Negative | **20** (48.8) |
| **Nodal metastases (n=21):** |  |
| Isolated tumour cells | **2** (5.9) |
| Micro | **8** (14.7) |
| Macro | **11**(29.4) |
| **Residual node count:** |  |
| 1-3 | **14** (35.3) |
| 4-10 | **6** (11.8) |
| >10 | **1** (2.9) |
| **Supplementary Table 2.** **Characteristics of residual tumour in those patients who did not achieve pathological complete response**. Samples insufficient for assessment were either too small (n=3), of inadequate quality (n=2) or were found to only have intravascular tumour emboli remaining in the breast (n=1). | |


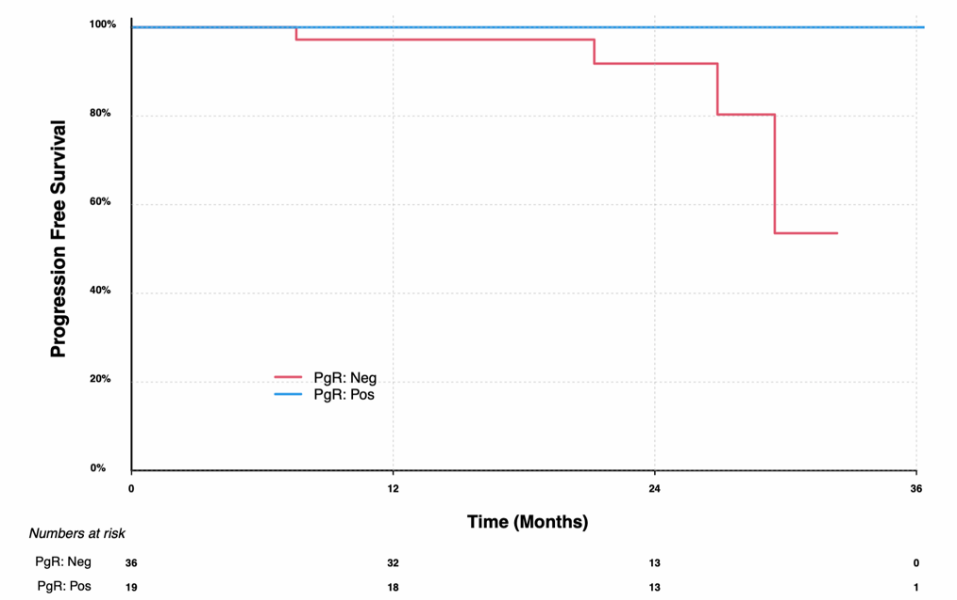


**A**

Estimated 2-year PFS: PgR positive tumours 100% (95%CI: 100-100%)

Estimated 2-year PFS: PgR negative tumours 92% (95%CI: 81-100%)


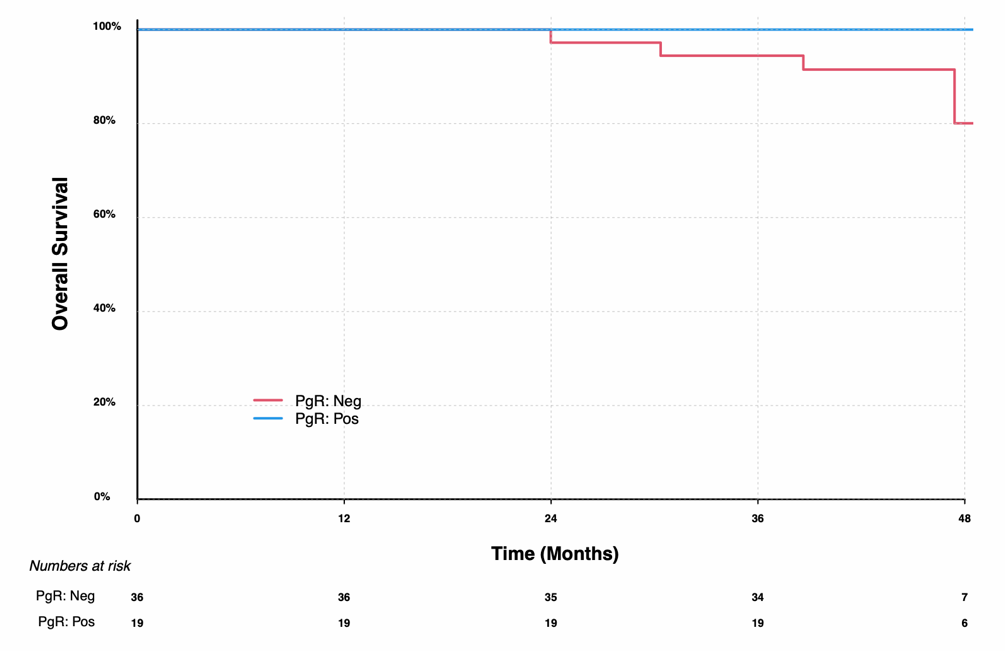


**B**

Estimated 2-year OS: PgR positive tumours 100% (95%CI: 100-100%)

Estimated 2-year OS: PgR negative tumours 97% (95%CI: 92-100%)

**Supplementary Figure 3. Progesterone receptor status outcome data.** Kaplan Meier curves demonstrating (A) progression free survival and (B) overall survival in PgR positive and PgR negative tumours. Estimated 2-year PFS and OS according to PgR status are provided below (hazard ratios and p-values were unobtainable for PgR data).


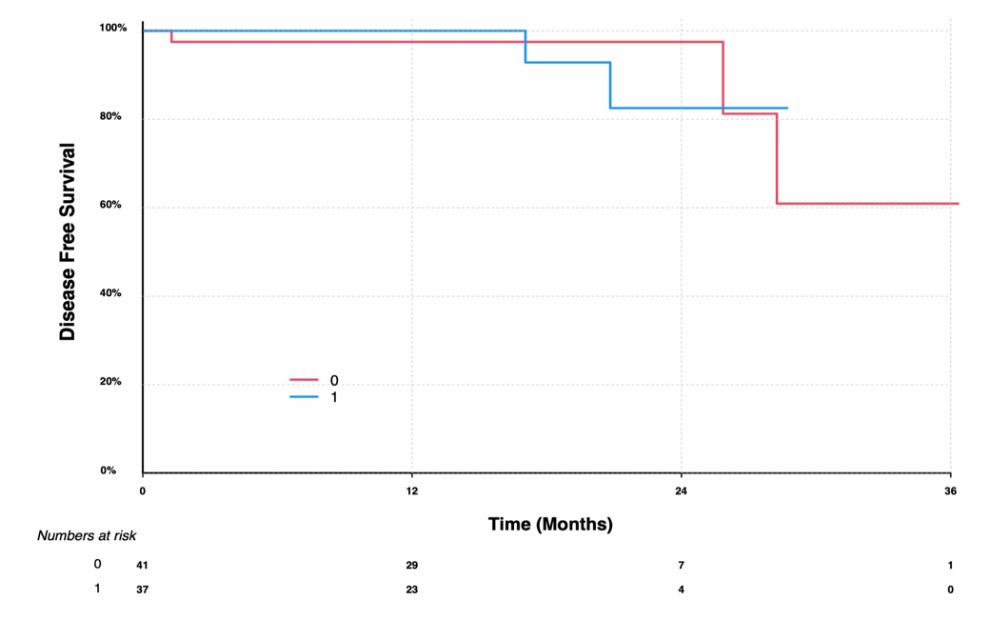


**A**

**No pCR**

**pCR**

Estimated 2-year DFS: pCR group: 83% (95%CI: 63-100%)

Estimated 2-year DFS: non-pCR group: 98% (95%CI: 93-100%) (HR: 1.5, 95%CI: 0.231-9.713, p= 0.672)


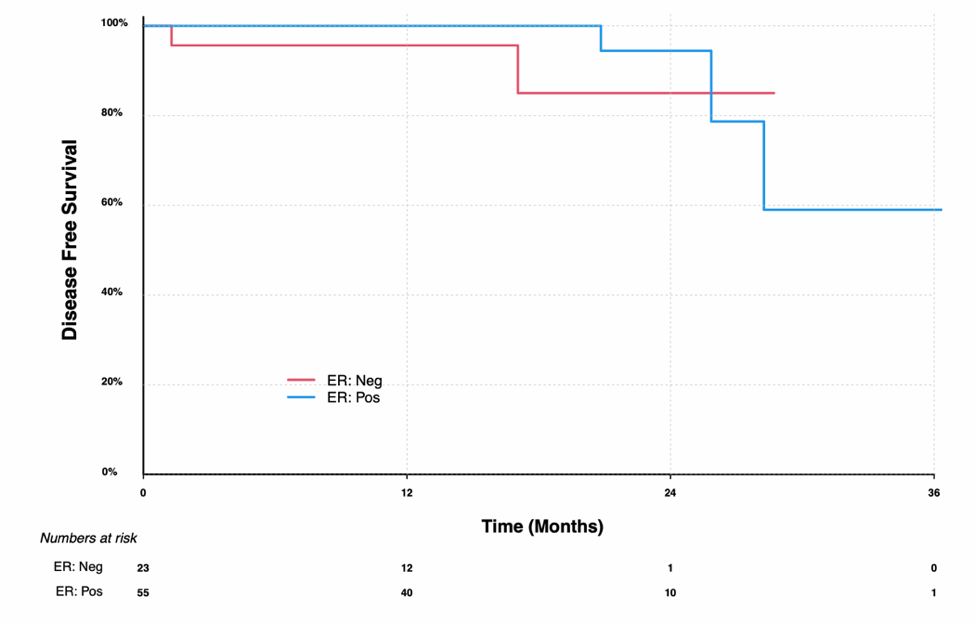


**B**

Estimated 2-year DFS: ER positive tumours 94% (95%CI: 84-100%)

Estimated 2-year DFS: ER negative tumours 85% (95%CI: 66-100%) (HR: 0.41, 95%CI: 0.067-2.509, p= 0.336)

**Supplementary Figure 4. Disease free survival Kaplan-Meier curves.** Disease free survival is stratified by (A) pCR status and (B) hormone receptor status. Estimated 2-year OS in each subgroup is provided with respective hazard ratios and p-values.


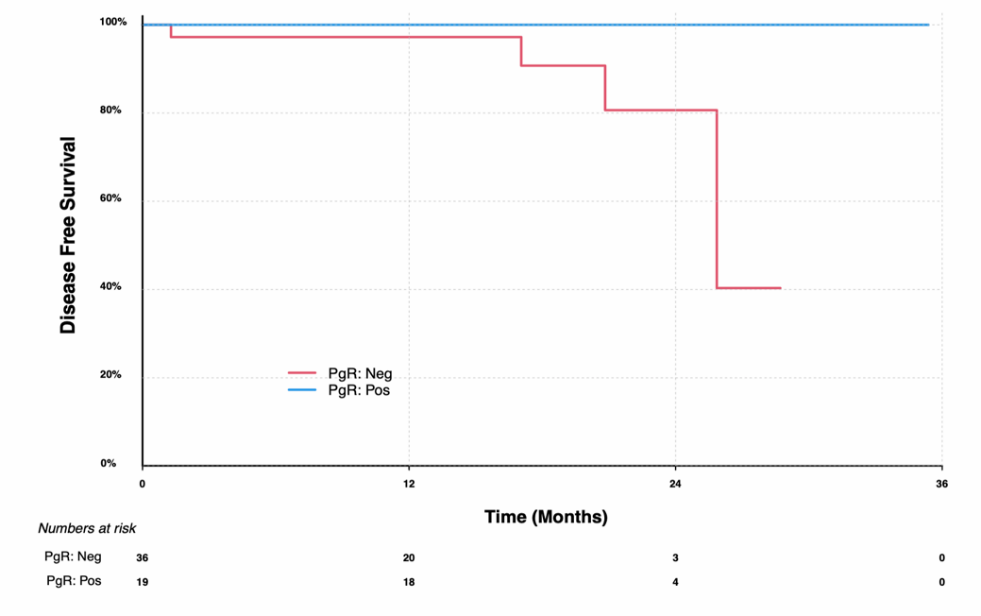


**C**

**C**

Estimated 2-year DFS: PgR positive tumours 100% (95%CI: 100-100%)

Estimated 2-year DFS: PgR negative tumours 81% (95%CI: 61-100%)

**Supplementary Figure 4. Disease free survival Kaplan-Meier curves.** Disease free survival is stratified by (A) pCR status (B) ER status and (C) PgR status. Estimated 2-year DFS in each subgroup is provided with respective hazard ratios and p-values (hazard ratios were unobtainable for PgR data).

| **Patient** | **pCR** | **Site of recurrence at initial relapse** | **Disease free survival (months)** | **Treatment** | | | | **Survival (months)*** |
| --- | --- | --- | --- | --- | --- | --- | --- | --- |
|  |  |  |  | 1 | 2 | 3 | 4 |  |
| 1 | N | Mastectomy scar | 1.3 | TDM-1 | Gemcitabine + carboplatin | Capecitabine | Eribulin | 17.7 |
| **2** | N | Cerebellum | 20.8 | Resection + cavity radiotherapy | n/a | n/a | n/a | 24.1 |
| **3** | Y | Right lung, hilar nodes, mediastinal nodes | 21.2 | TDM-1 | Nab-paclitaxel | Eribulin | Capecitabine | 38.6 |
| **4** | N | chest wall**,liver, vertebra | 25.8 | Paclitaxel | Capecitabine | n/a | n/a | 47.3 |
| **5** | N | Brain; later meningeal | 33.4 | Whole brain RT | Weekly paclitaxel; adjuvant trastuzumab/pertuzumab | n/a | n/a | 41.4 |
| **6** | Y | Brain, liver, bone | 43.9 | Whole brain RT | n/a | n/a | n/a | 46.5 |
| **Supplementary Table 3. Summary of patients who developed recurrent disease and the treatments received;** *All patients have died; **Found to be triple negative on biopsy of recurrence; AC: Adriamycin, cyclophosphamide; RT; Radiotherapy | | | | | | | | |

| Toxicity | Trial (n= % reported) | | | |  |
| --- | --- | --- | --- | --- | --- |
|  | NEOSPHERE | TRYPHAENA | BERENICE | KRISTINE | Hall et al 2020 |
| **Alopecia** | 64% | 48.6% | not reported | 63% | 95.8% |
| **Diarrhoea** | 46% | 61.1% | 10.1% | 73.5% | 73.6% |
| **Febrile neutropenia** | 8% | 18.1% | 17.2% | 16% | Not available* |
| **Cardiotoxicity** | 0% | 0.4% | 1.5% | <1% | 0% |
| **Nausea** | 36% | 52.8% | 2.0% | 58% | 68.1% |
| **Mucositis** | 21% | 23.6% | 3.5% | 13% | 69.4% |
| **Supplementary Table 4. Adverse events reported by published neoadjuvant, dual HER2 blockade trials**.  Rates reported include adverse events of all severity. Rates reported in this paper are documented in the far-right column. *5.2% of patients in this cohort presented with either Grade 3 of 4 infection during their course of treatment. | | | | | |

| **Study** | **Cohort** | **Regimens** | **Overall pCR rate** | **Hormone receptor positive pCR rate** | **Hormone receptor negative pCR rate** | **Median follow-up** |
| --- | --- | --- | --- | --- | --- | --- |
| **Gonzalez-Santiago et al 2020** | n=243 | 1. Anthracyclines and taxanes plus HP 2. Single agent taxane plus HP 3. Platinum plus HP | 1) 71%  2) 59.3%  3) 48.6% | 1) 58.9%  2) 58.8%  £) 39.1% | 1) 86.4%  2) 60.0%  3) 66.7% | Not reported |
| **Minhao et al 2020** | n=72 | 1. TCHP 2. THP | 1. 76.1% 2. 61.5% | 50.0%  *(overall)** | 85.7%  *(overall)** | 8 months |
| **Diaz-Redondo et al 2019** | n=254 | 1. NAC plus H 2. NAC plus HP | 1. 39.0% 2. 61.0% | 1. NR 2. NR | 1. NR 2. NR | Not reported |
| **Murthy et al 2018** | n=170 | 1. Taxane alone plus HP 2. anthracycline containing regimen plus HP 3. Carboplatin containing regimen plus HP | 1. 74% 2. 62% 3. 48% | 51%  *(overall)** | 71%  *(overall)** | Not reported |
| **Fasching et al 2018** | n=108 | 1. NAC plus HP 2. NAC plus H | 1. 52.8% 2. 30.2% | 1. NR 2. NR | 1. NR 2. NR | 60 months |
| **Spring et al 2018** | n=121 | 1. ACy-THP 2. TCHP 3. THP 4. ACy-TH | 1. 60% 2. 63% 3. 55% 4. 46% | 1. NR 2. 73.3% 3. 33.3% 4. NR | 1. NR 2. NR 3. 66.7% 4. NR | Not reported |
| **Singh et al 2017** | n=57 | 1. AC-PaHP | 1. 72% | 1. 68% | 1. 85% | Not reported |
| **Supplementary Table 5.** **Summary of real-world studies examining efficacy of neoadjuvant chemotherapy and pertuzumab/trastuzumab doublet** *Only overall pCR rates were reported in some papers but were not stratified according to both hormone receptor status and treatment regimen. NAC: neoadjuvant chemotherapy (taxanes with or without anthracyclines; regimen not specified) A: doxorubicin C: carboplatin; H: trastuzumab; P: pertuzumab; T: docetaxel Cy: cyclophosphamide Pa: paclitaxel *NR*: results were not specifically reported | | | | | | |
